# Increasing creatine kinase activity protects against hypoxia / reoxygenation injury but not against anthracycline toxicity *in vitro*

**DOI:** 10.1371/journal.pone.0182994

**Published:** 2017-08-14

**Authors:** Sevasti Zervou, Hannah J. Whittington, Philip J. Ostrowski, Fang Cao, Jack Tyler, Hannah A. Lake, Stefan Neubauer, Craig A. Lygate

**Affiliations:** Division of Cardiovascular Medicine, Radcliffe Department of Medicine, University of Oxford,Wellcome Trust Centre for Human Genetics, Roosevelt Drive, Headington, United Kingdom; University of PECS Medical School, HUNGARY

## Abstract

The creatine kinase (CK) phosphagen system is fundamental to cellular energy homeostasis. Cardiomyocytes express three CK isoforms, namely the mitochondrial sarcomeric CKMT2 and the cytoplasmic CKM and CKB. We hypothesized that augmenting CK *in vitro* would preserve cell viability and function and sought to determine efficacy of the various isoforms. The open reading frame of each isoform was cloned into pcDNA3.1, followed by transfection and stable selection in human embryonic kidney cells (HEK293). CKMT2- CKM- and CKB-HEK293 cells had increased protein and total CK activity compared to non-transfected cells. Overexpressing any of the three CK isoforms reduced cell death in response to 18h hypoxia at 1% O_2_ followed by 2h re-oxygenation as assayed using propidium iodide: by 33% in CKMT2, 47% in CKM and 58% in CKB compared to non-transfected cells (*P*<0.05). Loading cells with creatine did not modify cell survival. Transient expression of CK isoforms in HL-1 cardiac cells elevated isoenzyme activity, but only CKMT2 over-expression protected against hypoxia (0.1% for 24h) and reoxygenation demonstrating 25% less cell death compared to non-transfected control (*P*<0.01). The same cells were not protected from doxorubicin toxicity (250nM for 48h), in contrast to the positive control. These findings support increased CK activity as protection against ischaemia-reperfusion injury, in particular, protection via CKMT2 in a cardiac-relevant cell line, which merits further investigation *in vivo*.

## Introduction

Maintaining cellular adenosine triphosphate (ATP) levels is fundamental to efficient energy homeostasis in cells with either high or fluctuating energy demands, such as cardiomyocytes [1]. Creatine kinase (CK) catalyzes the reversible transfer of a phosphoryl group from ATP to creatine to form phosphocreatine (PCr) and adenosine diphosphate (ADP). Consequently, CK has a crucial role in storing, buffering and transporting high energy phosphates from the mitochondria to sites of energy demand [2–4].

There are 3 isoforms of creatine kinase in cardiomyocytes, namely, the sarcomeric mitochondrial (CKMT2), muscle and brain (CKB). CKMT2 is active when it forms octamers within the mitochondrial intermembrane space, while CKM and CKB exists as dimers forming the cytosolic isoenzymes MM-CK, BB-CK and MB-CK. CKMT2 and MM-CK are most abundant, representing 35% and 67% of total CK activity respectively, while CKB isoforms are very low abundance in the heart (1–3%) [5]. The substrate of the CK reaction, creatine, is taken-up by cardiac cells from the bloodstream against a concentration gradient via a specific plasma membrane transporter [6, 7].

In cardiac physiology, numerous studies have elucidated the role of mitochondria in oxidative stress-induced cell death [8]. For example, accumulation of calcium during the ischaemic period, combined with generation of reactive oxygen species (ROS) during reperfusion, synergistically promote formation of the mitochondrial permeability transition pore (mPTP), with subsequent loss of mitochondrial membrane potential and ultimately cell death [9, 10].

Mitochondrial CK is thought to be important for maintaining mitochondrial morphology by stabilizing contact sites between inner and outer mitochondrial membranes. Impaired activity was associated with loss of mitochondrial membrane potential and apoptosis [11]. The impact of CKM and CKMT2 double knock-out is less severe in the intact mouse, but nevertheless, hearts from these animals accumulated more calcium during ischaemia and had impaired functional recovery upon reperfusion [12].

Previous studies have demonstrated a rapid and irreversible loss of CKMT2 commensurate with ischaemic duration in the intact rabbit heart, and this correlated with contractile dysfunction during reperfusion [13]. This likely reflects direct oxidative damage since both peroxynitrite and superoxide can cause dissociation of CKMT2 octamers and enzyme inactivation [14].

There are similarities with anthracycline toxicity. In the clinical setting, use of anthracyclines for cancer chemotherapy, such as doxorubicin, is limited by dose-dependent development of cardiotoxicity manifesting as left ventricular dysfunction, cellular apoptosis and irreversible chronic heart failure [15, 16]. The molecular mechanisms are multi-faceted, but include production of ROS and oxidative damage [17], and there is a well-documented cellular energetics component (reviewed in [17]). For example, creatine kinase (including CKMT2) may be a specific target for damage, with anthracyclines shown to impair CK activity and reduce PCr/ATP ratio leading to heart failure *in vivo* [18].

One obvious approach is to augment CK activity in order to offset the effects of such damage. Previous studies have overexpressed CKM in the murine heart, which increased CK flux *in vivo* and improved functional recovery following *ex vivo* ischemia/reperfusion [19]. The same CKM overexpression model has also been shown to protect against doxorubicin-induced cytotoxicity, preserving cardiac function and improving survival [20]. However, it is unknown whether other CK isoenzymes would offer similar, or better, cellular protection. Previous studies showed that liver-specific overexpression of CKMT2 *in vivo* protected mitochondria from cyclosporine A- induced swelling and from subsequent mitochondrial transition pore opening [21]. BB-CK overexpression in liver–an organ that lacks endogenous CK expression- provides improved ATP buffering, with beneficial consequences against hypoxic ischaemic damage [22].

Here we tested the hypothesis that augmentation of the creatine kinase system *in vitro* can alter cellular response to different types of oxidative stress, in the form of either simulated ischaemia/reperfusion, or exposure to doxorubicin. For this purpose, we used a stable expression system in HEK293 cells and transient expression in the cardiac HL-1 cell line, to interrogate whether augmenting mitochondrial CKMT2, and the cytosolic isoforms CKM and CKB would protect against cell death. This study describes new observations on CK-mediated protection against I/R injury and could guide *in vivo* strategies for CK augmentation in the heart.

## Materials and methods

### Cloning of expression vectors

The open reading frames (ORFs) of CK isoenzymes namely human CKMT2, CKM, and CKB were amplified by polymerase chain reaction (PCR) using Phusion^®^ High-Fidelity DNA polymerase (New England Biolabs, Hitchin, UK) and mouse heart cDNA as a template. Each of the sequences were amplified using RPC- purified oligonucleotides (Sigma-Genosys, Gillingham UK) (Table 1 for sequence) that contained a Kozak sequence, restriction site, the ORF and a C-terminal hemagglutinin (HA) tag for protein detection purposes. ORFs were ligated into either pcDNA3.1 (-) or (+) (Promega, Southampton, UK) using T4 DNA ligase (Life Technologies Paisley, UK). To amplify the new constructs, *E*.*coli* (5α; NEB) transformation was used and ampicillin selection, followed by inoculating small scale cultures (for mini preps) and large-scale towards maxi-preps (Qiagen, Manchester, UK). The resulting DNA was checked for quality and quantity by a Nanodrop (Agilent, Stockport, UK) and was then sequence-verified by diagnostic restriction digestion using restriction sites specific to each construct (all enzymes and reagents supplied from NEB). Sequencing of cloned plasmids was outsourced to Source BioScience, Oxford, UK. For this purpose, primers were designed to ensure that the entire insert, including both ligation sites, was covered by at least two separate reads. Sequencing traces were analyzed using 4Peaks Software (v. 1.7.2). Consensus sequences were constructed from the individual sequencing reads using Serial Cloner (v. 2.6) and subsequently checked for alignment against the predicted sequence.

**Table 1:** Oligonucleotides used in creation of constructs.

ORF	Oligo name	Oligonucleotide sequence
**CKMT2**	**CKMT2_5’**	5’GACC *AAGCTT* AAGAAGAAGGATGGCCAGTGCCTTCTCAAAGTTGCT3’
	**CKMT2_3’**	5’CTAG*TCTAGA*TCA**AAGAGCGTAATCTGGAACATCGTATGGGTA**CTTCCTGCCAAACTGAGGCA3’
**CKM**	**CKM_5’**	5’GGGG *CTAGC* GTCGACACCATGCCGTTCGGTAACACCCAC3’
	**CKM_3’**	5’GGC *CTCGAG*CTA**TGCGTAATCTGGAACATCGTATGGGTA**CTTCTGCGCGGGGATCATGT3’
**CKB**	**CKB_5’**	5’CCCC *GCTAGC* GTCGACACCATGCCCTTCTCCAACAGCCAT3’
	**CKB_3’**	5’CCG*AAGCTT*GGTACCTA**TGCGTAATCTGGAACATCGTATGGGTA**CTTCTGGGCCGGCATGAGGTC

The underlined regions are the Kozak sequences (5’ primers) and stop codons (3’ primers). The restriction sites used for cloning are in *italic*. The HA tag sequence (in bold) is different in the CKMT2 3’oligonucleotide, as a slightly shorter version of the HA tag sequence was used for CKM and CKB constructs. A silent substitution (underlined) was incorporated at the +12 position in the CKM forward primer, as the CKM sequence was predicted to cause primer hairpin formation at this site (C replaced by T, i.e. codon changed from GGC to GGT, both of which encode glycine).

### Cell lines

Expression constructs pcDNA3.1- CKMT2/CKM/CKB–HA were transfected transiently into Human Embryonic Kidney Cells (HEK293) using FUGENE^®^HD (Promega) according to manufacturer’s instructions. Expression levels of the transgenes were verified by qRT-PCR and compared to untransfected HEK293 cells.

Stable cell lines were created for each of CKMT2, CKM and CKB by selection using geneticin (G418) (Life Technologies), 48 h post-transient transfection of the three vectors that were linearised at the *Mfe*I site. Following expansion of G418-resistant colonies, HA expression levels were tested by immunoblotting in addition to CK enzymatic activity using electrophoresis (Helena Biosciences, Gateshead, UK). A stable cell line expressing green fluorescent protein (GFP) in HEK293 was used as a control in the simulated ischaemia/reoxygenation experiment (a kind gift by Dr Mark Crabtree University of Oxford).

HL-1 mouse atrial tumor-derived cardiomyocytes were obtained from the Claycomb laboratory [23], used up to passage number 50 and were plated onto 24-well culture plates at a density of 0.5x10^5^ cells/well on gelatin/fibronectin-coated wells for transient transfection.

### Protein extraction and expression work

Cells were lysed by ice-cold RIPA buffer (Sigma, Poole, UK) containing protease and phosphatase inhibitors (Roche, West Sussex UK) in the culture dishes as described before [24]. Following electrophoresis, primary antibody against HA was used to probe the protein blots, followed by appropriate secondary antibody (Horseradish peroxidase; HRP–conjugated anti-rat; DAKO, Ely UK) and chemiluminescence detection (GE Healthcare Little Chalfont UK) and imaging by the Bio-Rad system (Watford, UK). For normalization purposes, the blots were stripped of the primary antibody and re-probed for beta-tubulin as a loading control (Abcam Cambridge UK). For immunocytochemistry against CKMT2 and cytochrome C oxidase IV (COXIV), antibodies were used from Abcam (COXIV), Roche (HA) and 4’,6-diamidino-2-phenylindole (DAPI)-containing mounting media from DAKO.

### Determination of creatine uptake by high-pressure liquid chromatography (HPLC)

Total Creatine levels (Cr+PCr combined) were measured by HPLC using a method adapted from [25] after exposure to exogenous creatine monohydrate at 250μM for 24h as published before [24]. Here we report [Cr] values. The conditions were optimized by previous pilot experiments that included a range of incubation time-points (0-48h) and creatine concentrations (0-10mM).

### Simulated ischaemia/reperfusion

For HEK293 stable cell lines and control cell line GFP-HEK293, 3.5–4.5x10^5^ cells were plated out onto 24 well plates and 24h after plating they were subjected to 1% O_2_, in ischemic-mimetic buffer (adapted from [26])(composition in mM: 125 NaCl, 8 KCl, 1.2 KH_2_PO4, 1.25 MgSO4, 1.2 CaCl_2_, 6.25 NaHCO_3_, 5 Na-lactate, 20 HEPES; pH 6.6, pre-equilibrated at 1% O_2_ overnight) for 18 h, followed by 2h of re-oxygenation at 18% O_2_, 5% CO_2_ in normoxic buffer (110 NaCl, 4.7 KCl, 1.2 KH_2_PO4, 1.25 MgSO4, 1.2 CaCl_2_, 25 NaHCO3, 15 glucose, 20 HEPES; pH 7.4 equilibrated in 18% O_2_ for 18h). All plates included wells with 1μM rapamycin as a positive control previously shown to protect against cell death in HEK293 cells [27, 28]. Cells were collected as described above and re-suspended in phosphate buffered saline (Life Technologies) containing propidium iodide (PI). Percentage of dead propidium iodide (PI)-positive cells was measured using the 488nm filter on the CyAN™ ADP Analyser flow cytometer (Beckman Coulter, High Wycombe, UK) and then analyzed using CyAN™ ADP Summit 4.3 (Beckman Coulter, California, USA). Acquisition thresholds were set to select for cell population and single cells and a live/dead gate set to 5% using a no-PI treated sample. The total cell number threshold for flow cytometry was set at 10,000 cells. For HL-1 cells, optimal hypoxic conditions were at 0.1% for 24 h while reoxygenation was at 18% O_2_ for 2 h, similarly to the protocol used for HEK293 cells. The pan-caspase inhibitor Z-VAD-FMK (carbobenzoxy-valyl-alanyl-aspartyl-[O-methyl]- fluoromethylketone) (zVAD-FMK) (at 100μM) was added to the cells 24h before hypoxia.

### Doxorubicin challenge

HL-1 cells were used for this experiment, as it was shown that HEK293 cells do not take up Doxorubicin and the autofluorescence displayed by doxorubicin, overlaps with PI fluorescent spectrum during flow cytometry (see Method for HEK293 viability results). HL-1 were plated at 8x10^4^/well on 24 well plates previously coated with 0.02% (w/v) gelatin/fibronectin. The cell line could not be stably transfected to express CK vectors due to high death rates during the antibiotic selection process. The cells were transfected transiently with 5μg DNA using FUGENE^®^HD (Promega), with a 3:1 ratio between transfection reagent (μl):DNA(μg), according to manufacturer’s protocol. Following transient transfection, cells were exposed to 120nmoles Trolox (6-hydroxy-2,5,7,8-tetramethylchroman-2-carboxylic acid; Sigma) and 24 h later, to 250nM doxorubicin (Sigma) for another 48h. Sham-transfected samples were included as controls and a GFP (green fluorescent protein) construct was co-transfected into HL-1 with CK constructs, for estimating transfection efficiency by a Nexcelom Cellometer (Nexcelom Biosciences, Massachusetts USA). At the end of the protocol, cells were detached from the wells using trypsin and centrifuged at 1,000 rpm for 5 minutes and then washed twice in phosphate buffered saline (PBS) and incubated with Annexin-Fluorescein isothiocyanate **(**FITC) (BioLegend, London UK) and Violet Live/Dead Dye (Thermo Fischer, Hemel Hempstead UK) for 30 minutes at room temperature at 1.25%, according to manufacturer’s instructions. Annexin V staining corresponds to reversible apoptosis and cells that have their membrane still intact. For this reason the total alive cell population includes the ANNEXIN-positive cells. Cell pellets were then analyzed by flow cytometry on a Beckman Coulter CyAn™ ADP flow cytometer, using the cyan (405nm) and violet (488nm) filters. 10,000 cells were collected from each sample. Analysis of flow cytometry data was conducted on Summit V4.3 as described above, and statistical analysis of treatment groups across 3 independent experiments, was conducted in Graphpad Prism 5.0.

### CK isoenzyme activity

3.5x10^5^ cells/well were plated onto 24 well plates and 24h later they were detached by trypsin and pellets were prepared as above, before re-suspending in ice buffer (0.08mM K_2_HPO4, 1mM EGTA, 0.02mM KH_2_PO_4_, and 1mM β-mercaptoethanol). Cells from 3 wells were pooled together for this purpose, using 1ml buffer. Samples were homogenized and made permeable using 0.1% v/v Triton X-100 (Sigma). Samples were stored on ice for at least 30 minutes to allow salt and debris precipitation and the supernatant was used for all subsequent enzyme activity measurements. Sample supernatant was diluted 1:200 in ice buffer and incubated with 1% CK isoenzyme activator for 10 minutes prior to use. Creatine kinase isoenzymes were separated according to their electrophoretic mobility on an agarose gel, followed by incubation with CK isoenzyme chromogen which allowed for visualization of the bands. Relative activities of individual CK isoenzymes were quantified by densitometry. All reagents were provided within the SAS-1 CK VIS-12 Isoenzyme kit, Helena Biosciences. Absolute activities for each isoenzyme were calculated by multiplying relative isoenzyme activity by total CK activity.

### Total creatine kinase activity

20μl of neat cell lysate (in ice buffer) was incubated with 1ml of CK-NAC reagent (Thermo Fisher Scientific) at 30°C. After a 3 minute lag time, CK activity was quantified using coupled enzyme reaction (CK-NAC, Helena Biosciences) and spectrophotometry [2](Biochrom Ultrospec 2100 Pro; Biochrom Ltd Cambridge, UK) by measuring the increase in absorbance at 340nm over 2 minutes as a result of reduced form of Nicotinamide adenine dinucleotide (NADH) production and analyzed using Swift Kinetics software v. 2.02 (Swift Analytical Ltd, York UK). The assay was performed in triplicate and results normalized to total protein levels using a BCA assay (Thermo Firsher).

### Statistical analysis

For group analysis (Simulated ischaemia/reperfusion,and Dox) the different groups were analyzed by one-way analysis of variance (ANOVA) and either Dunnett’s post-hoc test against the control group, Bonferroni post-hoc multiple comparisons test between all groups or between selected groups or unpaired Student’s t test (Graphpad Prism V5). At least three independent assays were performed per experimental section in this study and at least n = 3 replicates were included per treatment. Statistical significance was defined by *P* values <0.05.

## Results

### Characterization of stable cell lines

**Fig 1:**
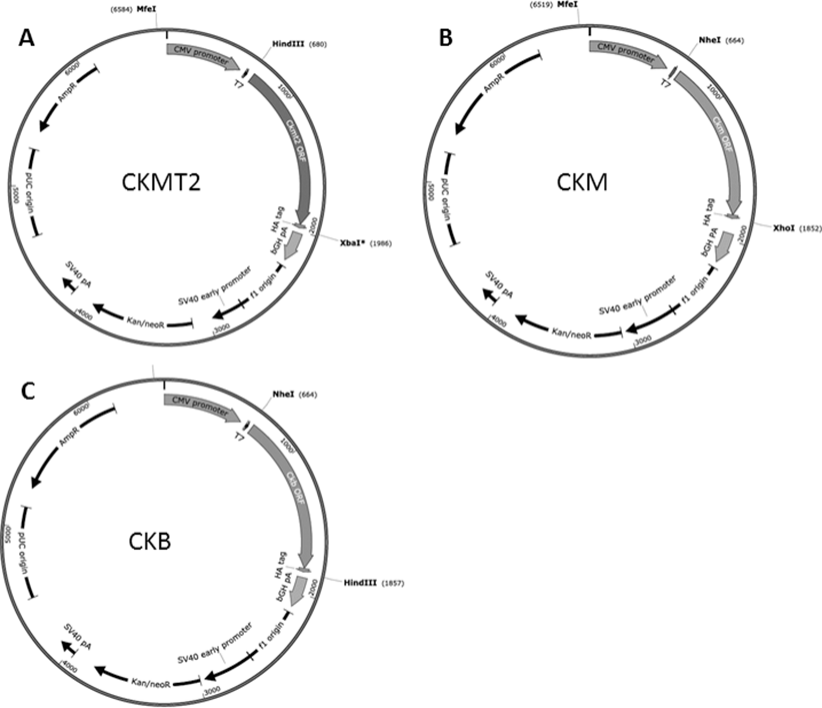
Cloning of human CK isoenzyme ORF sequences into pcDNA3.1(-). A-C: Each ORF was ligated into the multiple cloning site of the expression vector and in particular, for CKMT2 using *HindIII/XbaI*; for CKM, at the *NheI/XhoI* sites; for CKB at the *NheI/HindIII* sites. These restriction sites were unique in the final vector sequence. For protein detection purposes, all CK sequences were HA-tagged at the 3’ end. The three vectors were linearized at the *MfeI* site prior to transfection and stable selection by G418.

**Fig 2:**
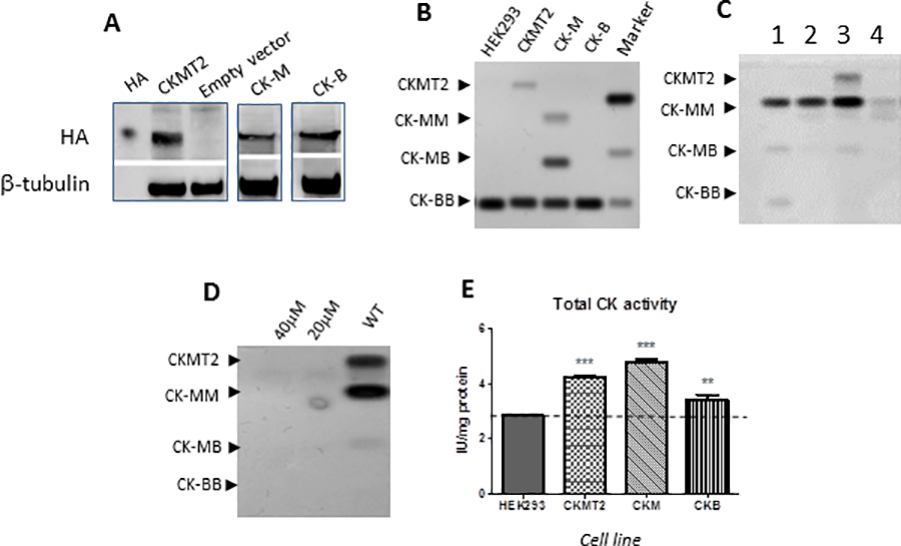
Stable cell line characterization. A. Immunoblotting determined the presence of the HA tag (‘HA’ positive control lane is included). Beta-tubulin was used as a loading control. Black boxes are present where lanes were not run continuously. B. Electrophoresis of CK isoenzymes in all cell lines, namely untransfected HEK293 in addition to CKMT2 (Mitochondrial Creatine Kinase)-, CKM (Muscle Creatine Kinase)- and CKB (Brain Creatine Kinase)-expressing HEK293 cells, followed by a marker lane. Arrows show respective CK isoenzyme bands. CKM migrates slightly faster in HEK293 cells compared to marker. C. Verifying specificity of SAS1 electrophoresis system. Marker (lane 1) in addition to: CKMT2 knockout (KO) heart sample (lane 2), lacking CKMT2 activity; a wild-type (lane 3); a double CKMT2 and CK-M KO (CKd KO), lacking CKMT2 and displaying traces of CK-MM (lane 4). D. DFNB, an inhibitor of CK activity added at 20 and 40 μM. E. Total CK activity in stable CK overexpressing cells was elevated compared to untransfected HEK293. Horizontal axis shows HEK293 untransfected or stable cell type. Data is mean ± SEM. One-way ANOVA and Dunnett’s post-hoc test against HEK293 values. *P<0.05; **P<0.01; ***P<0.001.

Three different expression constructs were created by cloning the full ORF of human CKMT2, CKM and CKB into pcDNA3.1(-) vector. Initial verification work confirmed the presence of the inserts following cloning and sequencing (maps are shown on Fig 1A–1C). Following stable selection with G418 the HEK293 cells overexpressing CK isoforms were tested for transgene expression levels and CK activity. CKMT2-, CKM- or CKB-HA protein was present in transfected cells and absent from control empty vector-transfected cells (Fig 2A). Beta-tubulin was used for protein loading purposes and was present in all samples analyzed.

Furthermore, basal CK activity was tested in HEK293 and HEK293-CKMT2,- CKM, -CKB (Fig 2B) using protein electrophoresis. All three CK isoenzymes were represented by protein bands on the gel and compared to known controls (marker). CK-BB is the most abundant isoenzyme in HEK293 cells shown by higher band intensity at the lower part of the gel. A CK control is included in the electrophoresis which has a CKM, CKMB and CKBB bands. CKMT2 migrates cathodically (opposite direction) to the rest of the isoenzymes, located at the top part of the gel. Overexpression of each of the CK isoenzymes was consistent with enhanced signal of the corresponding band. The isoenzyme electrophoresis tool was verified for band accuracy by analyzing samples that lacked CK [29], compared to previously published isoenzyme electrophoresis data (Fig 2C) or following inhibition of CK by 2,4-dinitro-1-fluorobenzene (DNFB) in samples prior to analysis [30](Fig 2D). While CKMT2 from mouse heart samples migrates at the expected size with reference to marker, in cells there is a slight deviation. CKMT2, CKM and CKB cells had elevated total CK activity (*P*<0.001 for CKMT2 and CKM; *P*<0.01 for CKB; One-way ANOVA, Dunnett’s post-test vs HEK293) (Fig 2E).

To test whether extra substrate in the form of exogenous creatine alters cell response against oxidative stress in synergy with overexpression of CK isoenzymes, all cell lines were exposed to 250μM to 10mM creatine monohydrate for 24hr. Both untransfected and stably transfected HEK293 were able to accumulate creatine, as tested by HPLC. During optimization work, 24 hours of creatine pre-exposure, increased intracellular creatine levels from 55±5nmol/ml to 66.7±4nmol/ml in HEK293 and from 58±4 to 84nmol/ml in CK–stably transfected cells.

### Stable CK overexpression protects against hypoxia/reoxygenation in HEK293 cells

**Fig 3:**
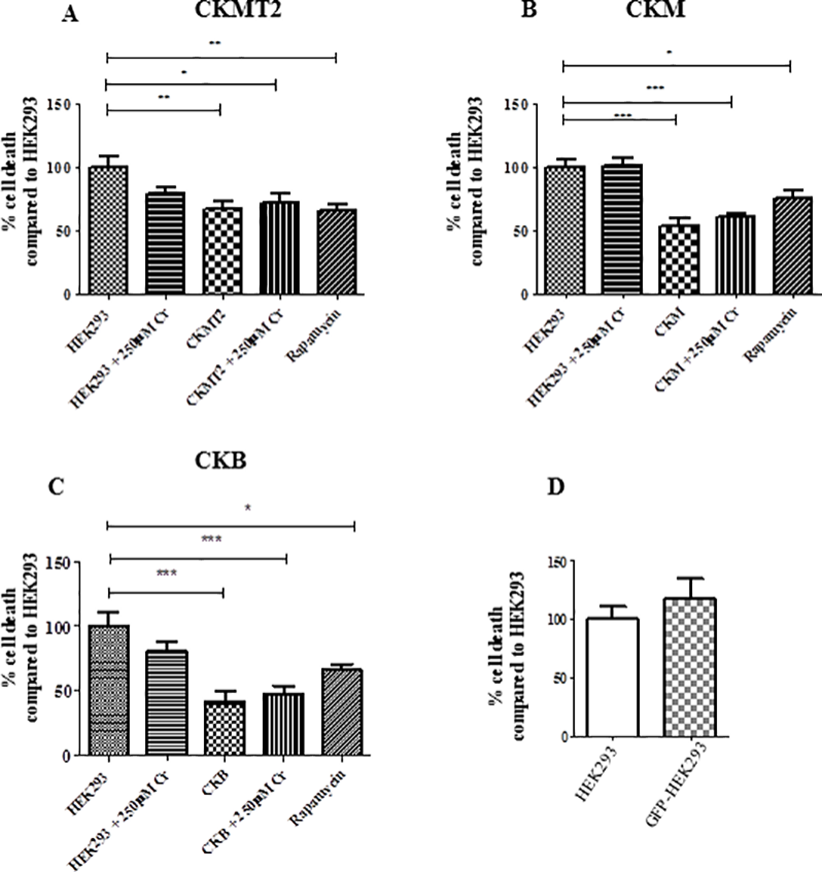
Simulated ischaemia/reperfusion *in vitro*. HEK293 and stable CK-expressing cells were exposed to 1% O_2_ for 18h in ischaemic media, followed by 2h re-oxygenation at 18% oxygen, prior to analysis for cell viability by propidium iodide incorporation. A. CKMT2 B. CKM C. CKB cells, all compared to HEK293 in the presence or absence of 250μM creatine monohydrate. Rapamycin (1μM) pre-exposure was used a positive control that preserves cell viability. Data are shown from at least 5 individual experiments and analyzed by One-Way ANOVA, Dunnett’s post-hoc test vs HEK293. ***P<0.001; **P<0.01; *P<0.05. D. GFP-HEK293 control cells were compared to HEK293 and showed lower survival rates (**P<0.01; Student’s t test) in contrast to the CK-stable cells in A-C. D. Control HEK293-GFP cell line showed similar death % to HEK293 (Student’s t test *P*>0.05) tested during n = 3 independent experiments. Data is mean ± SEM.

In an assay of ischaemia/re-oxygenation, stable overexpression of any one of the 3 CK isoforms was found to be protective, significantly reducing cell death when compared to untransfected HEK293 control cells. Cell death was lower by 33% in CKMT2 (*P*<0.01), 47% in CKM (*P*<0.001) and 58% in CKB (*P*<0.001) overexpressing cells (one-way ANOVA with Dunnett’s post-hoc test vs HEK293; Fig 3A–3C). Loading cells with creatine did not significantly reduce cell death in control cells, nor did it have an additive effect in CK overexpressing cells, although the protective effect of CK was recapitulated. In all assays, pre-incubation with rapamycin was used as a positive control and reduced cell death by 25–34% below HEK293 levels.

HEK293 cells stably transfected with GFP (GFP-HEK293) were used as a negative control to test whether creation of a stable cell line per se influences cell survival, e.g. by selecting for the most robust cells. No significant difference in cell death was observed compared to control untransfected HEK293 tested across 3 independent experiments (117%±17 vs 99%±11, unpaired Student’s t test P = 0.45; Fig 3D). This indicates that protection against hypoxia/reoxygenation in the CK stable cell lines was attributed to increased CK activity rather than off-target effects caused by the stable selection process.

### CKMT2 overexpression protects against hypoxia/reoxygenation in cardiac HL-1 cells

**Fig 4:**
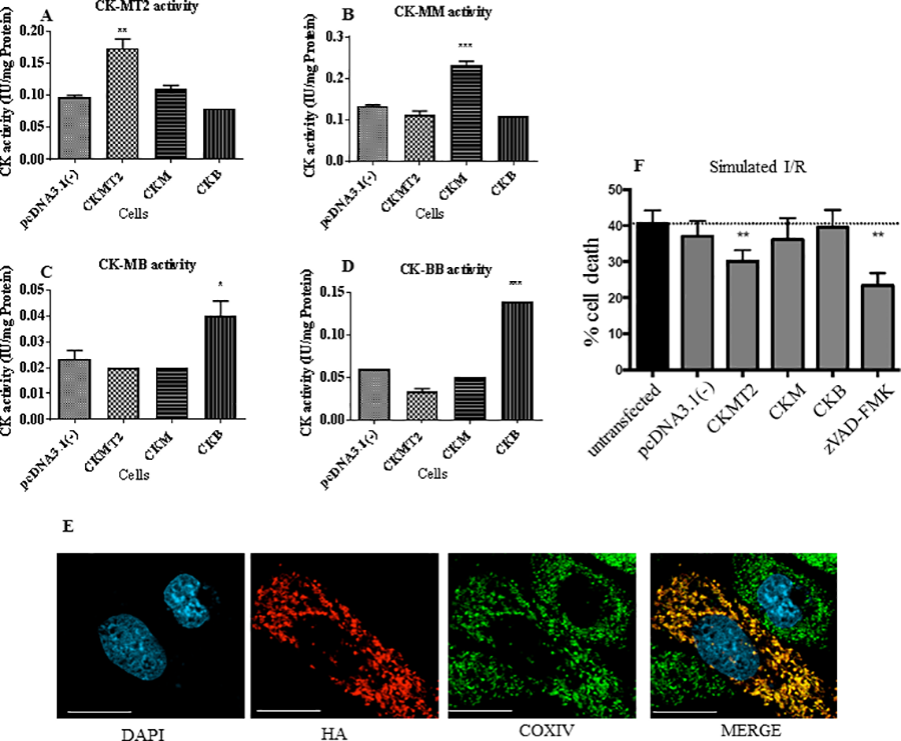
Simulated ischemia reperfusion in HL-1 cells. A-D: CK isoenzyme activity levels 48 h post-transient transfection of pcDNA3.1(-) or pcDNA3.1(-)-CK constructs. The horizontal axis shows the transgene and each graph corresponds to individual isoenzymes CK-MT2/MM/MB/BB respectively. E. Co-localization of CKMT2 and COXIV in the mitochondria in HL-1 cells, using confocal microscopy (Zeiss). Verification of HA-(CKMT2) expression in transfected cells and co-localization with mitochondrial COXIV staining. Images were acquired by confocal microscopy, using a 40x air objective. Blue corresponds to DAPI (nuclei); Red: HA tag; green corresponds to COXIV; Yellow is the overlap of COXIV and CKMT2 within mitochondria (MERGE). White scale bar: 20μm. F. Cell viability post-hypoxia-reoxygenation. Data represents results of six consecutive assays (n = 2 wells per treatment, per assay), compared to cell viability in untransfected controls for each assay, where untransfected and pcDNA3.1(-) levels are similar. The positive control caspase inhibitor zVAD-FMK was included per assay. Data is mean ± standard error of the mean (SEM). Results were analyzed by one-way ANOVA with Dunnett’s post-hoc comparison test vs pcDNA3.1(-)(CK activity) or vs untransfected (for I/R). *P<0.05; **P<0.01 ***P<0.001.

Following the positive effects of CK overexpression in HEK293 stable cell lines, the same experiment was repeated in cardiac atrial HL-1 cells. After transient transfection with either empty pcDNA3.1(-) or various CK isoform constructs as before, the CK activity was found to be significantly increased in an isoform-specific manner CKMT2 (*P*<0.001), CK-MM (*P*<0.001), and for the CKB construct, both CK-MB (*P*<0.05) and CK-BB (*P*<0.001) (Fig 4A to 4D). CKMT2 is the only isoenzyme expected to have mitochondrial localization, and this was confirmed by co-localization with the mitochondrial marker COXIV in the intermembrane space using immunocytochemistry (Fig 4E).

Compared to cell death in untransfected controls (40.5 ± 3.7%), there was no significant difference in cells transfected with empty vector (37.0 ± 4.2%), CKM (36.2 ± 5.9%), or CKB (39.5 ± 4.8%). However, transient transfection with CKMT2 caused a significant decrease in cell death (30.1 ± 3.1% compared to 40.5 ± 3.7% in untransfected; *P*<0.01). The caspase inhibitor zVAD-FMK was used as a positive control and also significantly protected HL-1 cells against cell death (23.4 ± 3.4%; *P*<0.01; Fig 4F).

### CK overexpression does not protect against doxorubicin-mediated cell death

**Fig 5:**
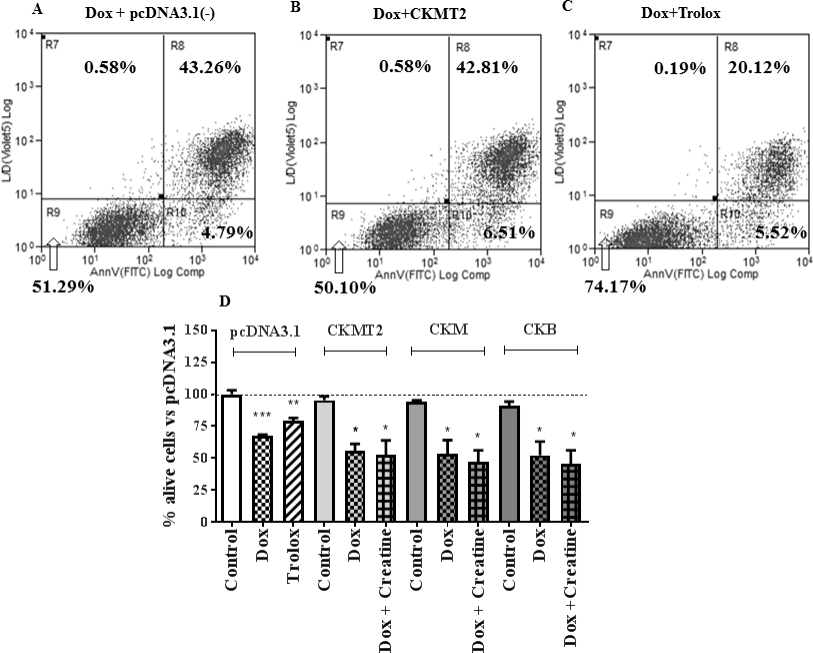
Representative example cytograms of HL-1 cells transiently transfected with empty vector pcDNA3.1 (A) or CKMT2 (B) transgene or pcDNA3.1 and pre-treated with positive control ROS scavenger Trolox (C). Percentage alive cells were measured via double staining with Live/Dead Violet stain (alive/dead cells) and Annexin V/FITC stain (apoptotic cells). Lower left quadrant (R9) represents % viable cells, which are negative for both dyes. Lower right quadrant (R10) includes % early apoptotic cells, positive for Annexin V/FITC stain only. Upper right quadrant (R8) shows % late apoptotic or necrotic cells, which stain both dyes. The graph on panel (C) shows data expressed as % compared to pcDNA 3.1 Control group (untreated). Treatment groups were compared to Control per cell type (One-Way ANOVA, Dunnett’s post-test and significance values are shown here. *P<0.05; **P<0.01; ***P<0.001. Trolox was protective against cell death One way ANOVA and Bonferroni post-test *P*<0.01). Data is mean ± SEM. Across cell types, Doxorubicin causes the same levels of cell death (One-Way ANOVA Dunnet’s vs pcDNA3.1).

HL-1 cells were transiently transfected to overexpress CK-pcDNA3.1 vectors as before and transfection efficiency was evaluated in each experiment by flow cytometry in cells co-transfected with GFP and was found to be 36% ± 2 across n = 3 assays. The protocol was optimized for a 48 hour, 250 nM doxorubicin insult (see Methods). Doxorubicin resulted in higher cell death in all cell lines compared to their respective controls but to a similar extent, as analyzed by one-way ANOVA and Dunnett’s post-test vs Doxorubicin in the pcDNA3.1 group (Fig 5D). The percentage of live cells includes all ANNEXIN-positive (reversibly apoptotic) cells since they had intact plasma membranes. In terms of ANNEXIN-positive signal, no differences were found in the number of apoptotic cells across all cell types, and mean ± SEM values were: pcDNA3.1: 5.7% ± 1; CKMT2: 5.7% ± 0.7; CKM: 5% ± 0.5; CKB: 5.4% ± 0.3, of total cell number, respectively.

Addition of creatine pre-treatment had no noticeable effect on survival rates for any of the CK isoenzyme-transfected cells (Fig 5D). In contrast, Trolox successfully protected the cells throughout the experiments serving as a positive control (79 ± 2 vs. 67 ± 2% alive cells; *P*<0.01 One-way ANOVA Bonferroni comparing Trolox to Doxorubicin groups).

## Discussion

In the current study, we used cell-based systems of CK isoenzyme overexpression to enhance total CK activity and investigate cell survival in response to different types of oxidative stress. HEK293 cells stably transfected with either CKMT2, CKM or CKB were protected against simulated ischaemia/reperfusion injury in the form of improved cell survival following hypoxia and re-oxygenation. However, when this experiment was reiterated in cardiac-derived HL-1 cells using transient overexpression, only the CKMT2 isoform provided effective protection.

There are a number of potential mechanisms that may explain the protective effect of CKMT2 overexpression. Most obvious, is the increase in CK enzymatic activity that was observed with both stable and transient transfection. This is likely to increase the capacity for regeneration of PCr at the onset of reoxygenation, which is associated with improved contractile recovery during reperfusion in mouse models of augmented energetics [2, 19].

A second potential mechanism is related to the structural role that CKMT2 fulfils by maintaining contact points between inner and outer mitochondrial membranes, thereby promoting mitochondrial integrity [31]. Increasing contact points, e.g. by overexpression of mitochondrial CK or hexokinase 2, has been shown to reduce probability of mPTP opening and cell death [32, 33]. In our transiently transfected HL-1 cells, we confirmed that transgenic CKMT2 protein was indeed located in the mitochondrial membrane as expected. This localization is via phospholipid attachment to mitochondrial membranes and ensures that CKMT2 is functionally coupled to oxidative phosphorylation via the adenine nucleotide translocase (ANT) and regulated by the outer membrane porin. Maintaining these protein interactions are not just important for effective phosphotransfer, but inhibition of mPTP opening by CKMT2 relies on the structural interaction between CKMT2 and ANT [34]. Relevant to this, is the high susceptibility that CKMT2 shows to damage by reactive oxygen species [14]. Oxidative stress can destabilize CKMT2 octamers to form less active dimers, leading to mPTP opening, apoptosis or necrosis [35] It is plausible that overexpression of CKMT2 may preserve the native enzyme, thereby maintaining functionality and providing relative protection.

Increasing intracellular creatine levels has also been shown to inhibit mPTP opening *in vitro* [2], and mito-CK localization within the inter-mitochondrial membrane is obligate for this effect [32]. Furthermore, mice with augmented cardiac creatine are less susceptible to ischaemia reperfusion injury *in vivo* [2]. It is therefore notable that the current study did not observe any additive effect of pre-loading cells with creatine. This implies, either, that there is sufficient substrate already present in HEK293 and HL-1 cells, or, that the energetic mechanism is not of primary importance.

A key question is why all CK isoforms protected HEK293 cells from hypoxia/reoxygenation, but only the mitochondrial isoform was protective in HL-1 cells, despite increased CK enzymatic activity in all. Firstly, this may simply be a gene dosing effect. We were not able to generate stable HL-1 cells, hence this work was performed following transient transfection and involved lower expression levels compared to the stable HEK293 cells. A second potential explanation may lie with differences in endogenous CK levels, since we found much higher CK activity in control HEK293 compared to control HL-1 cells. It is possible that pre-existing endogenous activity will make the cell more receptive to CK overexpression due to the presence of partner proteins, necessary for functional coupling of phospho-transfer. In this context, it is notable that functional coupling is deficient in HL-1 cells, precisely because there is low CK expression and poor organization of energy units [36]. Alternatively, low endogenous expression may favor a disproportionate response to CK overexpression. In this regard, control HEK cells express high levels of CKB, but negligible mito- and M-CK activity [37], which may favor a positive response from these isoforms. Similarly, augmenting the low levels of CKMT2 in HL-1 cells may provide the structural anchoring (as described earlier) to form functional energetic units that are otherwise missing.

Perhaps of greater concern is that CKM overexpression in mouse heart has already been shown to improve functional recovery following ischaemia / reperfusion *ex vivo* [19]. This is in contrast to our findings where CKM was protective in HEK293, but not in the cardiomyocyte-like HL-1 cells. So does this represent a false negative and to what extent can we trust the *in vitro* assays to meaningfully predict *in vivo* cardio-protection? This issue speaks to the inherent limitations of all *in vitro* experiments, which are reductionist by definition. However, care should be taken not to conflate very early recovery of contractile function (*in vivo*) with a read-out of cell death several hours later (*in vitro*), and it is notable that late myocardial injury was not assessed in CKM overexpressing mice. Regardless, we would argue that our cell-based system benefits from testing all isoenzymes in more than one cell line and allows us to effectively rate the most promising strategy for further protection studies. This would suggest that CKMT2 is the most robust and therefore strongest candidate to take forward *in vivo*.

We also tested whether CK overexpression protects against doxorubicin-induced cell death, which has been mechanistically linked to oxidative damage as well as energetic compromise [16, 18]. None of the CK isoforms modified survival rates following exposure of transiently transfected HL-1 cells to doxorubicin for 48 hours. Again, this is in disagreement with published data for cardiac CKM overexpression in mice, which exhibited improved survival and preserved CK flux and contractile function *in vivo* [20]. This calls into question the predictive value of the *in vitro* assay and there are multiple factors that may influence this. The issue of gene dosing in transient cells and the limitations of HL-1 cells as discussed above, are also pertinent here. In addition, HL-1 cells proliferate in culture and may therefore be more susceptible to DNA intercalation by doxorubicin compared to non-proliferative cardiomyocytes *in vivo*.

Previous studies in HL-1 and rat neonatal cardiomyocytes have shown that subclinical levels of doxorubicin cause ROS production and reduced creatine transport [38], and that elevating intracellular creatine protects against doxorubicin-induced cell death. [39] In contrast, we did not detect a beneficial effect of creatine loading in our assay and this may reflect the relatively high dose and longer pre-incubation time compared to Santacruz *et al*., i.e. 250μM for 48 h versus 1.5μM for 24 h. Our cellular insult may simply be too severe for energetic approaches to influence survival and it is notable that our positive control has an antioxidant mode of action.

Finally, the cascade of events leading to doxorubicin toxicity is likely to differ between an acute (*in vitro*) setting, compared to chronic 8 week exposure in a mouse model, or the clinical scenario in patients, where cardiac dysfunction may take years to manifest. In this context, we can only conclude that elevating CK isoforms does not protect against early, acute, doxorubicin toxicity and further studies are merited to determine the effect of CKB and CKMT2 on chronic exposure.

Our work exposes some of the limitations of *in vitro* assays, but nevertheless has strength in the comparison of various CK isoforms in more than one cell type. In particular, we present novel data suggesting that overexpression of mitochondrial CK may be beneficial in the setting of cardiac ischaemia / reperfusion injury, which will guide our future investigations *in vivo*.
